# Biodegradation of Pesticides at the Limit: Kinetics and Microbial Substrate Use at Low Concentrations

**DOI:** 10.3389/fmicb.2020.02107

**Published:** 2020-08-27

**Authors:** Johannes Wirsching, Holger Pagel, Franziska Ditterich, Marie Uksa, Martina Werneburg, Christian Zwiener, Doreen Berner, Ellen Kandeler, Christian Poll

**Affiliations:** ^1^Department of Soil Biology, Institute of Soil Science and Land Evaluation, University of Hohenheim, Stuttgart, Germany; ^2^Department of Soil Physics, Institute of Soil Science and Land Evaluation, University of Hohenheim, Stuttgart, Germany; ^3^Department of Environmental Analytical Chemistry, Institute of Applied Geoscience, University of Tübingen, Tübingen, Germany

**Keywords:** soil, low pesticide concentrations, biodegradation kinetics, functional gene abundance, gene transcription

## Abstract

The objective of our study was to test whether limited microbial degradation at low pesticide concentrations could explain the discrepancy between overall degradability demonstrated in laboratory tests and their actual persistence in the environment. Studies on pesticide degradation are often performed using unrealistically high application rates seldom found in natural environments. Nevertheless, biodegradation rates determined for higher pesticide doses cannot necessarily be extrapolated to lower concentrations. In this context, we wanted to (i) compare the kinetics of pesticide degradation at different concentrations in arable land and (ii) clarify whether there is a concentration threshold below which the expression of the functional genes involved in the degradation pathway is inhibited without further pesticide degradation taking place. We set up an incubation experiment for four weeks using 14C-ring labeled 2-methyl-4-chlorophenoxyacetic acid (MCPA) as a model compound in concentrations from 30 to 20,000 μg kg^–1^ soil. To quantify the abundance of putative microorganisms involved in MCPA degradation and their degradation activity, tfdA gene copy numbers (DNA) and transcripts (mRNA) were determined by quantitative real-time PCR. Mineralization dynamics of MCPA derived-C were analyzed by monitoring 14CO_2_ production and 14C assimilation by soil microorganisms. We identified two different concentration thresholds for growth and activity with respect to MCPA degradation using tfdA gene and mRNA transcript abundance as growth and activity indices, respectively. The tfdA gene expression started to increase between 1,000 and 5,000 μg MCPA kg^–1^ dry soil, but an actual increase in tfdA sequences could only be determined at a concentration of 20,000 μg. Accordingly, we observed a clear shift from catabolic to anabolic utilization of MCPA-derived C in the concentration range of 1,000 to 5,000 μg kg^–1^. Concentrations ≥1,000 μg kg^–1^ were mainly associated with delayed mineralization, while concentrations ≤1,000 μg kg^–1^ showed rapid absolute dissipation. The persistence of pesticides at low concentrations cannot, therefore, be explained by the absence of functional gene expression. Nevertheless, significant differences in the degradation kinetics of MCPA between low and high pesticide concentrations illustrate the need for studies investigating pesticide degradation at environmentally relevant concentrations.

## Introduction

Pesticide application is the dominant pest control method utilized by farmers in Germany, with an average application rate of 2.8 kg ha^–1^ yr^–1^ on arable land (Baumgarten et al., 2008). However, the behavior and ecotoxicological effects of many pesticides in the environment have not been adequately clarified (Sarmah et al., 2004; Carvalho, 2017). After application of pesticides, a considerable fraction ends up in the soil, where filtering functions, such as immobilization by clay minerals and organic matter (Sun et al., 2010), chemical and microbial degradation provide important ecosystem services for groundwater and surface water protection (Keesstra et al., 2012). Despite pesticide biodegradation, which is considered as the most important degradation pathway (Nowak et al., 2011), remnants of multiple pesticides (including their metabolites), persist at low but detectable and environmentally relevant concentrations in soils. As a result, soils shift from serving as sinks of pesticides to secondary sources (Miglioranza et al., 2002).

Silva et al. (2019) discovered that only 17% of 317 tested agricultural topsoils in the European Union (EU) contained no pesticides. While 25% of soils contained specific pesticide residues, 58% contained a mixture of several pesticides in medium and maximum concentrations of 0.02 to 0.04 mg kg^–1^ and 0.31 to 0.41 mg kg^–1^, respectively (Silva et al., 2019).

There is growing evidence that numerous organic pollutants persist in soil, even though they are fully biodegradable under optimal laboratory conditions (e.g., 2,4-dichlorophenoxyacetic acid; Nowak et al., 2011; Fenner et al., 2013). Processes effective in the field can be elusive at the laboratory scale. For example, pesticide residues may be bound to soil organic matter (SOM), especially due to the formation of hydrophobic SOM particles (Goebel et al., 2011; Kästner et al., 2014), making the pesticide inaccessible to microbes. If the pesticide is bioavailable and degraders are present, diffusion may limit microbial uptake of a compound (Dechesne et al., 2010). However, microbial pesticide decomposers may not be present or the heterogeneous distribution of pesticides and their degraders may result in low degradation rates (Dechesne et al., 2010). Pesticide molecules in inactive zones will be degraded either only in the case of new colonization by microbes or by mass transfer to adjacent zones (Dechesne et al., 2010).

The selected concentration range can also result in a difference between measured total mineralization in laboratory experiments and the fate of pesticide residues in the field. Most studies focus on relatively high concentrations, always with the maximum permitted application amount in mind, which ranges from 0.5 mg to 50 mg kg^–1^ (Jensen et al., 2004; Baelum et al., 2006; Jacobsen et al., 2008; Nicolaisen et al., 2008; Rosenbom et al., 2014). These concentrations, however, exist only immediately after application or occur in the event of accidental spillage resulting in local point infiltration (Helweg et al., 1998).

Currently, the microbial degradation of higher pesticide concentrations is well characterized. Degradation is often associated with the stimulation of microbial growth and metabolic mineralization, leading to the typical sigmoidal form of the degradation curves with a significant delay, exponential increase and saturation phase (Fomsgaard, 1997). This process can be described by a logistic function including a zero and first-order term in which, at some point, substrate availability becomes the limiting factor, and the curves begin to asymptote (Brunner and Focht, 1984; Fomsgaard and Kristensen, 1999). However, this process of degradation requires microbial utilization of pesticides as both energy and carbon sources (Duo-Sen and Shui-Ming, 1987). In contrast, fewer pesticide degradation studies in the range of 40–80 μg kg^–1^ have been published (Mueller et al., 1992; Helweg, 1993; Helweg et al., 1998). Fomsgaard (1997) demonstrated that degradation rates observed at higher concentrations cannot necessarily be transferred to degradation behavior at lower concentrations.

Fundamentally different preconditions influence microbial degradation at low pesticide concentrations. When pesticide concentrations are low, the distance between the molecule and the microorganisms increases, minimizing the probability of contact (Coche et al., 2018). Degradation of low pesticide concentrations often follows first-order kinetics (Jacobsen and Pederson, 1992; Helweg et al., 1998) without microbial uptake as an energy or carbon source (Fomsgaard and Kristensen, 1999). This lack of energetic use of pesticides at low concentrations may explain their incomplete microbial degradation. At low concentrations, the trade-off between gaining energy from the degradation of organic compounds and the energy and resources required for the expression of specialized enzymes involved in the degradation pathway could become negative. Microbes may also not be able to break down the pesticide, not because of energy limitations, but because no growth substrate (C) is available at the required concentration. Consequently, as concentrations of pesticides fall below a certain threshold, the growth of degrading microbes as well as the expression of related functional genes may be restricted (Egli, 2010).

One approach to address this topic is the use of model compounds such as 2-methyl-4-chlorophenoxyacetic acid (MCPA), as used in our study. Their degradation pathways and the protocols for detection of the relevant functional genes are well-known. The purpose of our study was to test whether the absence of functional gene expression under conditions of gradual energy or C limitation can explain the formation of pesticide residues in soil. We also wanted to correlate dynamic gene expression with alterations in MCPA degradation kinetics. We hypothesized that (1) degradation is impeded as soon as available pesticide concentrations fall below a threshold value that triggers the expression of relevant functional genes, (2) there are two different concentration thresholds for initiation of activity and growth of the microorganisms involved in degradation, and (3) the shift in growth and activity of degrader organisms with decreasing pesticide concentration is directly linked to changes in their metabolic utilization of the pesticide. To test these hypotheses, we set up a microcosm experiment using ^14^C-ring MCPA at concentrations from 30 to 20,000 μg kg^–1^ soil. By quantifying the abundance of the *tfdA* gene coding for the enzyme of the initial degradation step in which MCPA is converted into 4-chloro-2-methylphenol (MCP), we obtained an indicator for changes in the abundance of microorganisms carrying the *tfd*-degradation pathway. Based on this indicator, we determined the threshold concentrations above which an increase in microbial abundance was possible (Baelum et al., 2006; Ditterich et al., 2013). In addition, we quantified *tfdA* gene expression (mRNA) as an activity index to clarify whether different thresholds for growth and gene expression exist. Furthermore, we monitored the ^14^C flux in CO_2_ and microbial biomass as an indicator of the shift between anabolic and catabolic use of MCPA, to derive microbial carbon use efficiency (CUE).

## Materials and Methods

### Study Site and Soil Sampling

Soil for the experiment was taken from a study site located in the middle of the Ammer catchment between Herrenberg and Tübingen (48°33′24.664′′, 8°52′31.259′′) in southwest Germany. The parent rock of the study site is composed of Muschelkalk dips below mudstones, dolomites, and thin coal beds of Lettenkeuper. These layers are covered by thick loess layers that enable intense agriculture. We took soil samples in June 2016 from an Ap horizon (0–5 cm) of a silty Luvisol (World Reference Base for Soil Resources, Table 1). The sampled soil was in a dry condition with volumetric water content of 10%. Pesticide application was restricted to chloridazon and metamitron; MCPA had not been applied previously at this site. After sampling, the soil was sieved (<2 mm), homogenized, and stored at −20°C.

**TABLE 1:** Chemical and physical soil properties.

Soil horizon	Depth	pH	*C*_*org.*_	Nitrogen	Phosphate	Sand	Silt	Clay
Ap	[cm]	[CaCl_2_]	[mg g^–1^]	[mg g^–1^]	[mg g^–1^]	[%]	[%]	[%]
	0–5	6.48	18.4	2.1	1.038	2.26	72.04	23.83

### Experimental Design

The experimental design consisted of eight MCPA concentration treatments with three replicates each (0, 30, 50, 100, 500, 1,000, 5,000, 20,000 μg kg^–1^ soil), resulting in 24 microcosms per set. We selected these concentrations after the aforementioned publication of Silva et al. (2019) to account for possible MCPA concentrations directly after application as well as a depth-dependent dilution. At a soil density of 1.2 g cm^–3^ and a recommended application rate of 2.0 kg ha^–1^, 16.6 mg kg^–1^ would be present in the first centimeter of soil almost immediately after application. If we neglect the convective transport of MCPA, e.g., via preferential flow pathways, a further average dilution to 1.6 mg kg^–1^ follows over the first ten centimeters.

Three sets (24 microcosm × three) were spiked with ^14^C-ring labeled MCPA to follow MCPA mineralization and utilization by soil microorganisms. These samples were destructively sampled after 8, 15, and 37 days. Additionally, one set of 24 microcosms contained only unlabeled MCPA, from which a series of subsamples for ^14^C-free RNA/DNA co-extraction and MCPA quantification was taken and stored at −80°C until analysis (Table 2). Final concentrations of ^14^C MCPA in the microcosms were adjusted by mixing 15 kBq of labeled MCPA (purity 99%, specific activity 50–60 mCi/mmol; BIOTREND Chemikalien GmbH, Germany) with increasing levels of unlabeled MCPA (analytical grade MCPA 99.2% purity, Sigma-Aldrich, Germany). Briefly, soil equivalent to 50 g dry soil was weighed into plastic cups and soil moisture was adjusted by using a mixture of MCPA, deionized water, and trace amounts of ^14^C-labeled MCPA, to a volumetric water content of 25%. Soil moisture was maintained throughout the test. After thorough mixing of the applied solution with the soil, the cores were placed in the microcosms, which were then made airtight and frozen at −20°C.

**TABLE 2:** Time schedule.

Schedule for sampling analysis	Sampling time
^14^C mineralization	0, 1, 3, 6, 8, 10, 13, 15, 17, 20, 22, 24, 27, 29, 31, 34, and 37 d
^14^C-C_mic_	8, 15, and 37 d
MCPA concentration	0, 5, 9, 14, 21, and 37 d
DNA/RNA measurements	0, 5, 9, 14, 21, and 37 d

### MCPA Dissipation

After thawing, 2 g was mixed with 10 ml of methanol/H*2*O_deion_ (1:1 by volume) and placed on a horizontal shaker for 10 min at 200 rev min^–1^. Samples were then incubated in a water bath at +50°C for 60 min. Finally, the samples were shaken again for 10 min at 200 rev min^–1^ and centrifuged at 2,500 *g* for 10 min. The supernatant was transferred to brown vials with a 0.45 μm syringe filter.

Prior to HPLC-QqQ-MS/MS analysis, extracts were sonicated and vortexed for 5 min. Using a 1,260 Infinity system from *Agilent Technologies*, 1 μl of sample was injected onto a reversed phase column (*Agilent* Poroshell 120 C18, 2.1 mm internal diameter, 100 mm length, 2.7 μm particle size) at a temperature of 40°C. MCPA was eluted isocratically within 5 min using 50% water and acetonitrile (both acidified with 0.1% formic acid) at a flow rate of 0.4 ml min^–1^. After chromatographic separation, MCPA was detected by tandem mass spectrometry using an *Agilent* 6490 iFunnel Triple Quadrupole (QqQ) instrument. The analyte was ionized by negative electrospray (ESI), applying 12 l min^–1^ sheath gas (N_2_) at 400°C, 16 l min^–1^ drying gas (N_2_) at 150°C, 30 psi nebulizer pressure, 4.2 kV capillary voltage, and 1.2 kV nozzle voltage. MS/MS experiments were conducted by MRM (Multi Reaction Monitoring) using N_2_ as collision gas and collision energy (CE) dependent mass transitions (MCPA: quantifier 198.9/140.9 at 10 eV, qualifier: 198.9/34.9 at 45 eV). The limit of quantification (LOQ) was defined at 13 μg kg^–1^ MCPA in soil.

### Measurement and Model-Based Evaluation of MCPA Mineralization

The ^14^CO_2_ evolution of the microcosms was determined via titration (DIN EN ISO 16072:2011-09). Vials attached to the undersides of the microcosm lids contained 2 mL of 1 M NaOH, which acted as a CO_2_ trap. The respiration rate was measured in an aliquot of 0.5 ml, which was treated with 0.5 ml of 1 M BaCl_2_ and two drops of phenolphthalein prior to being titrated against 0.1 M HCl. The neutralization reaction endpoint was indicated by a colorless, completely transparent solution. For analysis of ^14^CO_2_ from microbial respiration, 1 ml of NaOH from the microcosm was mixed with 4 ml scintillation vial fluid (Rotiszint Eco Plus, Carl Roth GmbH + Co. KG) in a 5 ml scintillation vial (LDPE). The decay rate in Becquerel (Bq) was measured on a scintillation counter (Wallac 1411, liquid scintillation counter, United States). To account for interfering substances, a quenching adaptation with ^14^C-aqueous standards was used to improve the accuracy of the actual counts per second () for the entire energy band.

To derive the half-life of MCPA mineralization for each concentration treatment, analytical solutions of a kinetic model (Eq. 1) based on Duo-Sen and Shui-Ming (1987) and extended by Fomsgaard and Kristensen (1999) were fitted to cumulative ^14^CO_2_ data. The reference value used to calculate the half-life refers to the maximum mineralizable ^14^C content in the soil. It does not include the immediate ^14^C assimilation by microorganisms.

Briefly, the full model describes the formation of ^14^CO_2_ by two processes: direct mineralization of MCPA and formation of microbial biomass (first term in Eq. 1) and first-order mineralization of MCPA-derived ^14^C that had been incorporated into SOM (second term in Eq. 1). The analytical solution of the full model reads as follows:

(1) $$C=C_{0}\left(1-\frac{k_{1}C_{0}}{\left(k_{1}+k^{*}C_{0}\right)e^{k_{1}t}-k^{*}C_{0}}\right)+S_{0}\left(1-e^{-k_{2}t}\right)$$

where *C* is MCPA-derived ^14^CO_2_ (% of initially applied MCPA), *C*_0_ is the total mineralizable MCPA (% of initially applied MCPA), *S*_0_ is the total mineralizable MCPA-derived ^14^C in SOM (% of initially applied MCPA), and *k*_1_ (d^–1^), *k*^∗^ (%^–1^ d^–1^) and *k*_2_ (d^–1^) are rate constants. For simplification and to provide meaningful parameter constraints for non-linear regression, we reparametrized the model by introducing the dimensionless parameter

(2) $$f_{k}=-\frac{k^{*}C_{0}}{k_{1}}$$

The range of *f*_*k*_ is constrained as 0 ≤ *f_*k*_* < 1 due to the given constrains of the original model that *k*^∗^ < 0, *k_1_* > 0, *C*_0_ > 0 and 1 + *k^∗^C*_0_/*k*_1_ > 0 (Duo-Sen and Shui-Ming, 1987). Substituting *f*_*k*_ into Eq. 1 eliminates *k*^∗^ and gives:

(3) $$\begin{array}{c} C=C_{0}\left(1-\frac{k_{1}C_{0}}{\left(k_{1}-f_{k}k_{1}\right)e^{k_{1}t}+f_{k}k_{1}}\right)+S_{0}\left(1-e^{-k_{2}t}\right)\\ C=C_{0}\left(1-\frac{C_{0}}{\left(1-f_{k}\right)e^{k_{1}t}+f_{k}}\right)+S_{0}\left(1-e^{-k_{2}t}\right) \end{array}$$

Parameter estimation was done by non-linear least squares regression with the Levenberg-Marquardt algorithm using multiple starting values as implemented in the R-package nls.multstart (Patfield and Matheson, 2008). Half-lives were calculated by numerically solving the corresponding non-linear equation derived from Eq. 3 using Newton’s method as implemented in the R-package *nleqslv*:

(4) $$0=e^{{}^{k_{2}T_{1/2}}}-\frac{e^{{}^{k_{1}T_{1/2}}}\left(2f_{k}S_{O}-2S_{0}\right)-2f_{k}S_{0}}{e^{{}^{k_{1}T_{1/2}}}\left(f_{k}\left(S_{0}+C_{0}\right)-S_{0}-C_{0}\right)-f_{k}\left(S_{0}+C_{0}\right)+2C_{0}}$$

Two simplified expressions of Eq. 3 were used if parameter estimates of the full model became highly uncertain and practically unidentifiable (as indicated by asymptotic confidence intervals):

(5) $$C=C_{0}e^{-k_{1}t}$$

(6) $$C=C_{0}\left(1-\frac{C_{0}}{\left(1-f_{k}\right)e^{k_{1}t}+f_{k}}\right)$$

Half-lives from estimated parameters of fitted Eqs 5 and 6 were calculated according to Eqs 7 and 8, respectively (see also Duo-Sen and Shui-Ming, 1987).

(7) $$T_{1/2}=\frac{\ln\left(2\right)}{k_{1}}$$

(8) $$T_{1/2}=\frac{1}{k_{1}}\ln\left[\frac{1}{1-f_{k}}+1\right]$$

Finally, the full model (Eq. 3) was fitted to ^14^CO_2_ mineralization data of experiments with 30–500 μg kg^–1^ initial MCPA, first-order kinetics (Eq. 5) was fitted to data of experiments with 1,000 and 5,000 μg kg^–1^ initial MCPA, and a model version neglecting incorporation of MCPA-derived ^14^C into SOM (Eq. 6) was fitted to the data of the experiment with 20,000 μg kg^–1^ initial MCPA. An R-script of model-based data evaluation of ^14^CO_2_ is provided as Supplementary Material.

### Microbial Biomass (*C*_mic_)

^14^C incorporation into microbial biomass carbon was determined using the chloroform fumigation-extraction (CFE) method adapted from Poll et al. (2010). In short, 10 g soil was fumigated with ethanol-free chloroform for 24 h. A second 10 g of the same soil was the non-fumigated control. To extract soil organic carbon, 40 ml of 0.5 M K_2_SO_4_ was added to each fumigated and non-fumigated sample. The samples were shaken at 200 rev min^–1^ on a horizontal shaker for 30 min and centrifuged at 4,400 *g*. The clear supernatant was filtered through a 20 μm filter and diluted 1:4 with deionized water to avoid high salinity during detection. The supernatant was measured for organic carbon using a total organic C analyzer (multi-N/C 2100S, Analytic Jena AG, Jena, Germany). The *C*_mic_ content was determined from the difference in C content between the fumigated and non-fumigated samples using a k_EC_ factor of 0.45 (Joergensen, 1996). To determine ^14^C content in *C*_mic_, 1 ml of the CFE supernatant was mixed with 4 ml scintillation vial fluid (Rotiszint Eco Plus, Carl Roth GmbH + Co. KG) in a 5 ml scintillation vial (LDPE). The calculation of incorporated ^14^C was carried out as described for *C*_mic_ content, using the difference in activity for the fumigated and non-fumigated samples, but here the undiluted supernatant was used.

### Measuring Carbon Use Efficiency (CUE)

Carbon use efficiency was calculated on the basis of a biomass-based method described by Manzoni et al. (2012), which can be applied to labeled substrate.

(9) $$CUE=\frac{14_{Cmic}}{14_{Cmic}+R_{cum}}$$

14_*Cmic*_ = ^14^C uptake in microbial biomass

*R*_*cum*_ = cumulative respiration rate.

### Molecular Analysis

RNA and DNA were extracted from 2 g frozen soil using the RNeasy PowerSoil Total RNA Kit for soil and the RNeasy PowerSoil DNA Elution Kit (Qiagen, Germany) in a co-extraction method following the manufacturer’s protocol. For further analyses, RNA and DNA samples were stored at −80°C.

Prior to reverse transcription, residual DNA in the RNA samples was digested with Turbo DNase (TURBO DNA-free^TM^ Kit, Invitrogen, Thermo Fisher Scientific, Germany; Table 3). Each reaction contained 20 μl RNA sample, 2.4 μl 10× Turbo DNA buffer and 1.6 μl Turbo DNase.

**TABLE 3:** Primers sequences and conditions for quantitative PCR.

Target sequence	Primer*	qPCR conditions	References
16S rRNA genes	341F: CCT ACG GGA GGC AGC AG 515R: ATT ACC GCG GCT GCT GGC A	600 s at 95°C, Cycle (35): 15 s at 95°C, 30 s at 60°C, 30 s at 72°C, and 30 s at 75°C	López-Gutiérrez et al. (2004)
*tfdA*	F: GAG CAC TAC GCR CTG AAY TCC CG R: GTC GCG TGC TCG AGA AG	600 s at 95°C, Cycle (40): 15 s at 95°C, 30 s at 64°C, 30 s at 72°C, and 30 s at 81°C	Baelum et al. (2006)

For reverse transcription, the SuperScript^TM^ III Reverse Transcriptase Kit with Random Primers, RNase OUT (Invitrogen, Thermo Fisher Scientific, Germany), and 10 mM dNTPs (Genaxxon, Germany) were used according to the manufacturer’s protocol and reaction conditions described in Supplementary Table S1. Two subsamples of each RNA extract were used: one subsample was treated with reverse transcriptase in the second reaction step (cDNA sample); the other was supplemented with nuclease-free water instead (RNA sample) to account for DNA residues.

Real-time PCR quantification was performed on all cDNA and RNA samples. The determination of total bacteria (16S rRNA genes) and MCPA degraders (total *tfdA* gene abundance) was carried out using an ABI Prism 7500 Fast (Applied Biosystems, Germany). qPCR conditions are described in Table 3. The qPCR efficiency ranged from 95 to 105%. The SYBR Green reaction was performed using the Power SYBR^®^ Green Kit (Applied Biosystems, Germany) according to Ditterich et al. (2013). Standard DNA was used for quantification with a dilution series from 10^8^ copies μl^–1^ to 10^1^ copies μl^–1^ for each standard. The detection limit was on the order of 10^3^ copies per g^–1^ dry weight.

### Statistical Analysis

To examine the influence of the different concentration treatments on MCPA dissipation, i.e., *tfdA* gene abundance and expression, we used a mixed-effect model with repeated measurements, taking into account time as a within-subject factor. MCPA treatment was implemented as a categorical predictor. The individual microcosms were considered as a random effect. The modeling function *lme* is included in the package “*nlme*” of the statistical software R (R Core Team, 2018). Since we did not want a pairwise comparison for all MCPA treatments, we specified comparisons of interest using linear contrasts. In that way, the intercept at the lowest concentration (30 μg kg^–1^ soil) was the average baseline across all levels. The comparison of parameters derived from the logistic model, such as half-life time, was carried out with Tukey’s honest significant difference test, included in the package “*emmeans.*” Test assumptions were checked visually by residual diagnostic plots (Kozak and Piepho, 2018). If two or more levels of a factor did not differ from each other, but did probably differ from another level, we unified the levels or “pooled” them together to achieve model simplification.

## Results

### MCPA Dissipation

**FIGURE 1:**
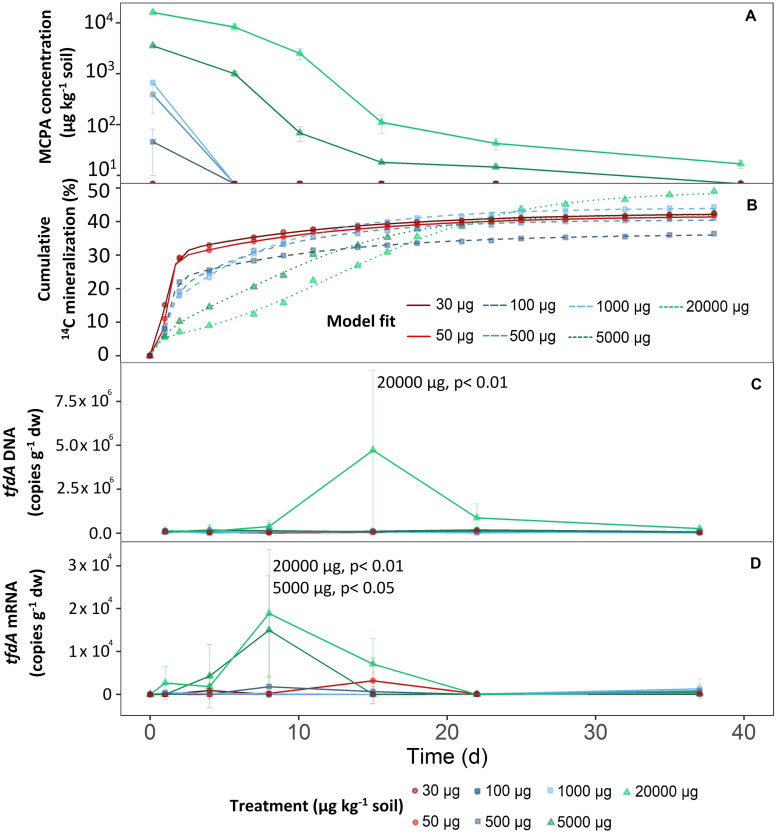
Mineralization of different MCPA concentrations as a function of time. **(A)** Decrease in MCPA concentration (detection limit, 13 μg kg^–1^ soil). **(B)** Mineralization of MCPA is represented by the percentage of initial ^14^C–MCPA. Curves were fitted to the data points via a logistic model. **(C)** Progress curves of tfdA gene copy numbers in relation to dry weight. **(D)** Progress curves of tfdA gene expression in relation to dry weight. Data are presented as means ± SD. Statistically significant differences represented as significance levels of *p* ≤ 0.05 and *p* ≤ 0.01 (*n* = 3).

2-Methyl-4-chlorophenoxyacetic acid dissipation was highly dependent on its initial concentration (Figure 1A). Based on MCPA dissipation, the concentration treatments could be divided into three groups.

The first group consisted of the two lowest concentration treatments; 30 and 50 μg kg^–1^. After only 1 day, extractable MCPA was below the detection limit (Figure 1A).

The mid-range group, consisting of concentration treatments from 100 to 1,000 μg kg^–1^, also showed rapid, absolute dissipation. After eight days, no MCPA could be extracted from the soil.

On day eight and compared to the concentration range of 100 to 1,000 μg kg^–1^, 28 and 51% of the initial concentrations of 5,000 and 20,000 μg kg^–1^ were still present, respectively. After 21 days at an initial concentration of 5,000 μg kg^–1^, 14.7 ± 1.4 μg kg^–1^ were still detectable, and after 36 days it was no longer possible to extract MCPA from the soil. For the highest MCPA treatment (20,000 μg kg^–1^), only 99.9% disappeared after 36 days, which meant that an absolute concentration of 16.7 ± 3.2 μg kg^–1^ remained in the soil.

### ^14^CO_2_ Mineralization and Half-Life Time (DT_50_)

Total mineralization (percentage of initial MCPA application), calculated according to Eq. 3, reached saturation in all treatments (Figure 1B). In the concentration range from 30 to 5,000 μg, the level of saturation was about 40% (Figure 1B).

In the case of the highest concentration only, MCPA mineralization rose to a significantly higher plateau, reaching approximately 50% of initial MCPA application (*C* = 51.57 ± 2.9; *F*_1_,_14_ = 15.30, *p* < 0.05).

**FIGURE 2:**
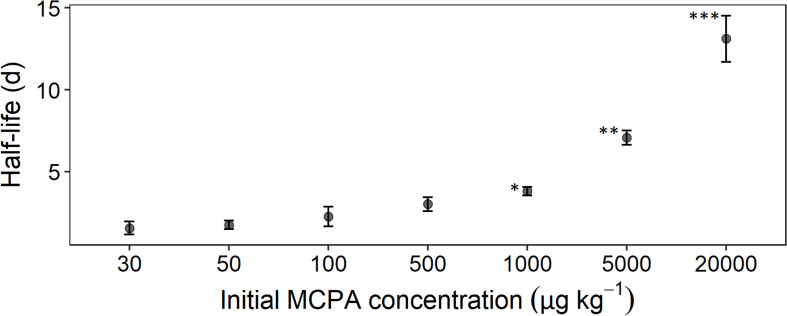
Half-life time as a function of initial concentration calculated according to Eq. 8. Error bars represent the standard deviation. The statistical comparison is always checked against the concentration treatment with 30 μg. Asterisks indicate *p*-values smaller than *p ≤ 0.05, **p ≤ 0.01, and ***p ≤ 0.001 (*n* = 3).

The mineralization rate, expressed as half-life time, reflected, to some extent, the absolute MCPA reduction. A rapid degradation rate for the concentration range from 30 to 500 μg kg^–1^ was seen in the half-life time of 2 to 3 days (Figure 2). From 1,000 μg and upward, a significant deceleration of mineralization was detectable. In the case of the two highest concentrations, this resulted in half-life times of 8 and 14 days, respectively.

### ^14^C Assimilation (^14^C-C_mic_) and Carbon Use Efficiency (CUE)

**FIGURE 3:**
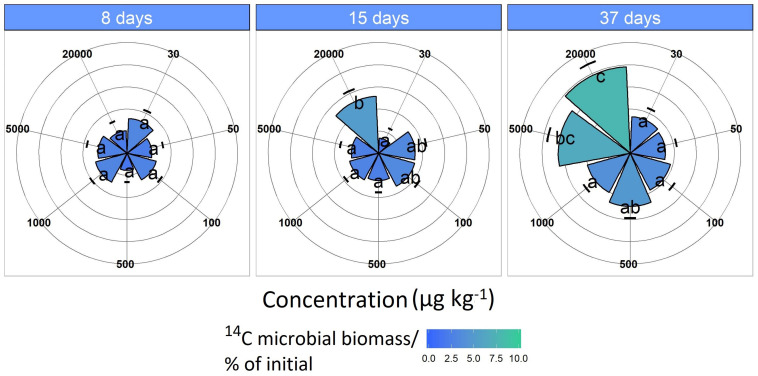
^14^C assimilation originates from the labeled MCPA as a function of time. Pie slices and the color gradient from blue to green represent the ^14^C assimilation for each concentration. Blue indicates a minor and green a higher ^14^C uptake. Data are presented as means ± SD. Letters indicate a statistically significant difference at *p* ≤ 0.05 (*n* = 3).

Microbial ^14^C uptake from the labeled MCPA increased only slightly for concentrations in the range of 30 to 1,000 μg kg^–1^ during the 37-day incubation period, remaining almost constant at 2 to 3% (Figure 3).

The MCPA treatment 20,000 μg kg^–1^ had significantly increased ^14^C incorporation after 15 days (*F*_1_,_118_ = 5.33, *p* < 0.05), which was approximately 7%, and this increased to about 10% as the experiment progressed (37 days; *F*_1_,_118_ = 19.9, *p* < 0.01).

**FIGURE 4:**
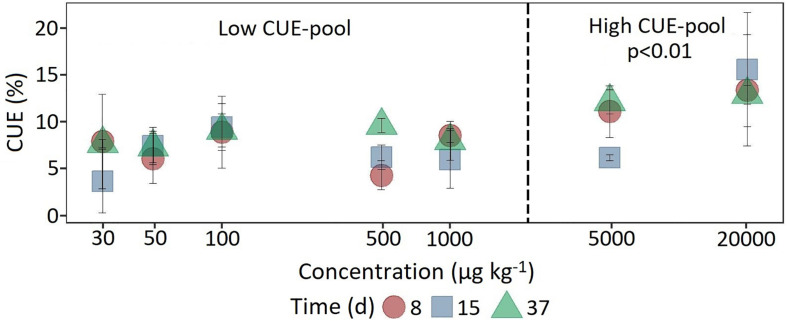
Dynamics of carbon use efficiency calculated from the ratio of respiration and ^14^C assimilation at the beginning, in the middle and at the end of the experiment as a function of concentration. For statistical comparison, low (30 to 1,000 μg kg^–1^ soil) and high (5,000 to 20,000 μg kg^–1^ soil) concentration levels are pooled together based on the shift in substrate utilization between 1,000 and 5,000 μg kg^–1^ soil. Statistically significant differences are represented as significance levels of *p* ≤ 0.01 (*n* = 3).

In comparison, at a concentration of 5,000 μg kg^–1^, a significant increase (*F*_1_,_118_ = 7.18, *p* < 0.05) in ^14^C content was observed only after 37 days (Figure 4). The CUE, calculated from the ratio of ^14^CO_2_ respiration and ^14^C-assimilation, was significantly higher for the two highest concentrations (CUE%, mean = 11.9 ± 4.32; *F*_1_,_61_ = 25.15, *p* < 0.01) compared to the lower concentrations (mean = 7.35 ± 2.71).

### *TfdA* Gene Abundance (*tfdA* DNA) and *tfdA* Transcript Abundance (*tfdA* mRNA)

There were no differences in microbial abundance (16S rRNA) between the individual treatments (data not shown). Only the highest concentration of 20,000 μg kg^–1^ significantly increased *tfdA* gene copy numbers (*F*_1_,_107_ = 14.98, *p* < 0.05). After a short adaptation phase, *tfdA* DNA increased steadily from the fifth day onward and reached a maximum of 4.73 × 10^6^ copy numbers g^–1^ after 15 days (Figure 1C). The fraction of *tfdA*-harboring microorganisms then decreased between the 15^th^ and 23^rd^ day as quickly as they had increased (Figure 1C).

Compared to the *tfdA* DNA, the *tfdA* mRNA transcript level was slightly more sensitive to MCPA addition. The concentration treatments of 5,000 (*F*_1_,_105_ = 3.39, *p* < 0,05) and 20,000 μg kg^–1^ (*F*_1_,_105_ = 14,29, *p* < 0,05) supported a significant increase in activity for the microorganisms involved in MCPA degradation compared to concentrations of less than 1,000 μg kg^–1^. In general, *tfdA* gene expression began 4 days before copy numbers of the *tfdA* gene increased and reached a maximum of 1.5 × 10^4^ and 1.89 × 10^4^ copy numbers per g dry weight for the concentrations 5,000 and 20,000 μg kg^–1^, respectively, after 8 days. Compared to the almost uniform copy number increase, activity decreased more rapidly at 5,000 μg kg^–1^ than at 20,000 μg kg^–1^; i.e., no further tfdA gene expression was detected after 15 days. For the concentration of 20,000 μg kg^–1^, *tfdA* mRNA abundance reached baseline levels after 22 days (Figure 1D).

## Discussion

### MCPA Dissipation and Cumulative ^14^CO_2_-Respiration

The persistence of pesticides at environmentally low concentrations has been rarely investigated, although their degradation kinetics may substantially differ from degradation at high concentrations (Fomsgaard, 1997). The aim of this study was, therefore, to identify shifts in degradation kinetics along a concentration gradient and to test whether the dynamics of the degrader population could explain the formation of persistent pesticide residues. Our initial hypothesis that MCPA degradation is impeded below a specific concentration threshold was not confirmed. However, we were able to group initial MCPA concentrations into two classes, with concentrations <1,000 μg kg^–1^ exhibiting rapid mineralization and concentrations >1,000 μg kg^–1^ exhibiting delayed mineralization.

Our observation that higher concentrations were associated mainly with delayed mineralization is consistent with previous studies (Helweg et al., 1998; Fomsgaard and Kristensen, 1999; Baelum et al., 2006; Johnsen et al., 2013). At concentrations of 5,000 and 20,000 μg kg^–1^, the lag phase was approximately 3 to 5 days (Figures 1A,B). Three main processes for the occurrence of delayed mineralization are mentioned in the literature:

(1) An unbalanced initial mRNA synthesis rate of *tfdA* and *tfdB* can lead to toxic 4-chloro-2-methylphenol levels in the cell at initial MCPA concentrations that are much higher (50 and 150 mg kg^–1^) than applied in our study (Leveau et al., 1999). The range of initial concentrations used in this study should, therefore, not result in significant metabolite concentrations (Jensen et al., 2004).
(2) According to Fomsgaard and Kristensen (1999) two sequential first order processes could also be responsible for an extended lag phase at higher concentrations. First, the dissolved fraction of the pesticide is degraded. This is subsequently followed by first-order degradation of the organic matter in which the sorbed fraction of the pesticide is incorporated (Fomsgaard and Kristensen, 1999). However, MCPA is anionic and therefore weakly adsorbed to soil colloids, usually with *K*_*d*_ in the range of 0.3–1 l kg^–1^ (Helweg, 1987; Socías-Viciana et al., 1999; Hiller et al., 2006). Sorption should therefore play a minor role.

The third, and in our case most probable, explanation, is that MCPA degradation was initially limited by the number of MCPA degraders. This presumption was supported by the increase in the number of *tfdA* genes (see below) and by the fitted modified logistic model of Duo-Sen and Shui-Ming (1987). In cases where growth was seen, the curves had a sigmoidal form, reflecting the logistic growth of microorganisms. Comparable results were reported in Reffstrup et al. (1998) where the mineralization of mecoprop concentrations of 5 to 500 mg kg^–1^ was described by an exponential form and low concentrations of 0.0005 to 0.5 mg kg^–1^ by the zero and first-order forms of the 3/2-order model.

Concentrations of 30 to 50 μg kg^–1^ showed no lag phase in MCPA mineralization, which contradicts our hypothesis that energy limitation occurs at low concentrations. An initial density of the *tfdA* gene harboring microorganisms in the range of 1.62 × 10^5^ copy numbers g^–1^ was sufficient to convert this MCPA concentration immediately after application. We found that MCPA extraction was no longer possible after a conversion of approximately 40% of the initial applied MCPA (Figures 1A,B), which is consistent with previous studies (Helweg, 1993; Helweg et al., 1998). According to Nowak et al. (2011), another fraction of the MCPA-C remains in the soil as biogenic residue, where degrading microorganisms utilize the carbon derived from the pollutant to form cellular components.

### ^14^C Assimilation (^14^C-C_mic_) and Carbon Use Efficiency (CUE)

The classification of the initial MCPA concentrations according to degradation kinetics was confirmed by differences in MCPA-derived ^14^C assimilation. At the two high MCPA concentrations, an initially limiting MCPA degrading population was able to grow by incorporating more ^14^C through higher CUE. Together with the modified fitted logistic model this indicates a shift from catabolic to anabolic microbial utilization of MCPA-derived carbon.

Transfer of the ^14^C label to microbial biomass is a useful tool for monitoring the formation of biogenic residues and deducing a total mass balance (Kästner et al., 2016). The difference between the original amount of MCPA-derived C minus its mineralization is often referred to as non-extractable residues (NER; Kästner et al., 2016). According to Nowak et al. (2011), a significant contribution of biogenic residues to NER formation can be expected, as long as the respective pesticide can be easily degraded by microbes with significant CO_2_ formation. In the present study, about 10% of MCPA-derived C remained in the soil as microbial biomass C. However, it is not possible to define exactly the origin of this ^14^C fraction, i.e., whether it is exclusively from active microorganisms involved in degradation or if a portion of the ^14^C has been recycled via cross feeding. Assuming that the ^14^C is present in microorganisms that are still active, this 10% is slightly below the 20% reported in the study by Nowak et al. (2011) in the case of 2,4-D, which nevertheless explains a considerable proportion of the mass balance. Consequently, for the concentration range from 5,000 to 20,000 μg kg^–1^ MCPA, the following estimate of the carbon distribution is obtained: about 50% respiration, 10% in the living biomass and 40% remaining as biogenic residues or NER.

In comparison, at concentrations below 1,000 μg, the low incorporation into living biomass (2–3%) indicated that energy use and microbial biomass build-up can also occur at low concentrations. The metabolic utilization of low MCPA concentrations is supported by a functional mechanism that is encoded in the *tfdK* gene and allows MCPA uptake against a concentration gradient; i.e., the concentration inside can be higher than that outside the cell, enabling the use of low pesticide concentrations (Leveau et al., 1998). Therefore, if an active transport system as for MCPA degradation exists, pesticides can probably be degraded even at micromolar concentrations.

### *TfdA* Gene (DNA) and Transcript Abundance (mRNA)

In accordance with our second hypothesis, we identified two different concentration limits for growth and activity that supported our classification of the initial MCPA concentration into groups with different residence times (Figure 2). To distinguish between growth and activity, the number of copies of the *tfdA* gene and transcription level of mRNA synthesis were used as growth and activity indices, respectively. The observed increase in activity at the two highest MCPA concentrations coincided with the slow onset of MCPA dissipation and preceded the actual growth of *tfdA*-harboring microorganisms. Interestingly, the maximum *tfdA* mRNA level was almost identical for both concentrations 5,000 and 20,000 μg kg^–1^ (1.5 × 10^4^ and 1.89 × 10^4^ copy numbers g^–1^, respectively). This observation is supported by the study of Leveau et al. (1999), in which the maximum values of mRNA showed no significant increase from 0.1 to 1 mM 2,4-D (0.1 and 1 mM correspond to 20,000 and 200,000 μg kg^–1^). Ledger et al. (2006) found that this could be explained by a downregulation of *tfdA* synthesis to avoid toxic intermediates. Another reason for similar synthesis maxima at two different initial concentrations may be identical cell concentrations carrying the *tfd*-degradation pathway at the beginning of MCPA degradation.

The actual differences of the higher MCPA concentrations between 5,000 and 20,000 μg kg^–1^ was due to the longer duration of activity, up to 8 days for the highest concentration treatment. This observation was also described in the study by Leveau et al. (1999), in which a 1 mM concentration shift led to a prolongation of *tfd* expression which could be attributed to a prior decrease in the remaining MCPA to below a threshold for mRNA synthesis at 5,000 μg kg^–1^.

The inability to detect an increase in *tfdA* gene copy numbers and transcription below 1,000 μg kg^–1^ can be attributed to several factors. On the one hand, the half-life of mRNA is on the order of minutes (Selinger et al., 2003), which means that the timing of measurement is crucial for detectability; mRNA formation may have peaked between MCPA application and the first sampling date. However, we assume that activation of mRNA synthesis requires more than 2 days, since the two highest MCPA concentrations showed a lag phase in mRNA synthesis. On the other hand, the high MCPA concentrations were associated with an increase in *tfdA* mRNA, underscoring the coupling of gene expression with the presence of MCPA (Leveau et al., 1999). In the case of low molar concentrations, this relationship between gene expression and MCPA concentration can be decoupled; low constitutive enzyme levels would lead to the direct conversion of a small amount of MCPA (Leveau et al., 1999). Due to the rapid degradation rate at concentrations below 1,000 μg kg^–1^, which could not be associated with additional *tfdA* expression to concentrations above the constituent level, our first hypothesis must be rejected.

However, we could not determine a concentration-related threshold below which the expression of relevant functional genes was inhibited and at which overall pesticide degradation did not continue. This is due to the aforementioned mechanisms that initiate immediate MCPA degradation. Active intracellular transport of low molar concentrations and constitutive gene expression together provide a degradation potential that is independent of the MCPA concentration in the soil.

## Conclusion

This study focused on the biodegradation of gradually increasing pesticide concentrations using a combination of ^14^C isotope analysis to model degradation kinetics, analysis of functional genes, and transcript abundance. We used MCPA, with a known degradation pathway, as a model compound and clearly showed that fast and slow MCPA degradation are controlled by the initial concentrations, and follow the pattern of physiological reactions. However, we had to reject our first hypothesis that below concentration threshold MCPA degradation is limited by the absence of functional gene expression.

We suggest that our results for MCPA could be extended to other compounds with low sorption affinities and uptake systems that permit transport into the cell at low molar concentrations. For components with higher sorption affinities than MCPA, different degradation mechanisms that determine fate at low concentrations may apply in soils. Further studies are therefore needed to investigate the behavior of moderately and highly sorptive pesticides at low molar concentrations. These should address the research questions of specific gene regulation in relation to the degradation of low pesticide concentrations and the influence of spatial distribution on bioavailability at low pesticide concentrations.

## Data Availability Statement

The raw data supporting the conclusions of this article will be made available by the authors, without undue reservation.

## Author Contributions

JW, EK, CP, HP, MU, and DB contributed to the conception and design of the study. JW performed the ^14^C mineralization, CO_2_ measurement and microbial biomass determination, supported by DB. JW performed the data evaluation and statistics. HP carried out the model fitting for half-life estimation and wrote the corresponding R-code. MU and FD developed the molecular biological methods. MW and CZ developed the measuring method for MCPA using LS-MS/MS. JW wrote the first draft of the manuscript. All authors contributed to the revision of the manuscript, read, and approved the submitted version.

## Conflict of Interest

The authors declare that the research was conducted in the absence of any commercial or financial relationships that could be construed as a potential conflict of interest.
