# Supplementary material for: Biodegradation of Pesticides at the Limit: Kinetics and Microbial Substrate Use at Low Concentrations
Source: Front Microbiol. 2020 Aug 27;11:2107. doi: 10.3389/fmicb.2020.02107 (PMC7481373; doi:10.3389/fmicb.2020.02107)
Supplement: Supplementary file 1 [file Table_1.DOCX]

# **Supplementary**

Supplementary Tabel 1: Reaction mixture and conditions for reverse transcription

| Reaction | mixture | Temperature profile |
| --- | --- | --- |
| reaction 1  (two preparations per sample) | 11 µl DNA digestion sample  1µl random primer  1 µl dNTPs | 5 min at 65°C  >= 1 min at 4°C |
| reaction 2  (for each sample) | 1. Preparation (+ samples):   4 µl 5x First-Strand Buffer  1 µl 0.1 M DTT (100mM)  1 µl RNase OUT  1 µl reverse transcriptase (200 U/μl)  13 µl reaction 1 | 5 min at 25°C  60 min at 50°C  15 min at 70°C  Cool-down at 4°C |
|  | 1. Preparation (- samples):   4 µl 5x First-Strand Buffer  1 µl 0.1 M DTT (100mM)  1 µl RNase OUT  1 µl DEPC water  13 µl reaction 1 |  |

**R code of the applied model**

require(ggplot2)

library(purrr)

library(dplyr)

library(tidyr)

library(broom)

library(nls.multstart)

library(nlstools)

library(nleqslv)

library(devtools)

library(ggpubr)

## Model after Duo-Sen, L. and Shui-Ming, Z. (1987)

# Kinetic model for degradative processes of pesticides in soil.

# Ecological Modelling 37, 131-138. doi:10.1016/0304-3800(87)90021-4

# set working directory

getwd()

setwd("E:/Campos/Campos/R Skript/Model_Holger")

HBW <-read.table("Halbwertzeit_statistik.csv",header = T, sep = ";" )

# read data

Halbwertzeit<-(Halbwertszeit_statistik)

head(dat)

# set factors

dat$rep<-as.factor(dat$rep)

dat$mcpa<-as.factor(dat$mcpa)

dat <-dat%>%

mutate(time_d = Incubation_time_hours/24)

write.table(pars_final, file = "Halbwertszeit.csv", sep = ";", col.names = NA,qmethod = "double")

# DEFINITION OF FITTING FUNCTIONS

f_full<-function(dat){

# fit over each set of groupings

fits <- dat %>%

group_by(., mcpa, rep) %>%

nest() %>%

mutate(fit = map(data, ~ nls_multstart(percent ~ C0-C0/((1-f_k)*exp(k1*time_d)+f_k)+S0*(1-exp(-k2*time_d)),

data = .x,

iter = 1000,

start_lower = c(C0=0, f_k=0, k1=0, S0=0, k2=0),

start_upper = c(C0=60, f_k=0.999, k1=10,S0=60,k2=10),

supp_errors = 'Y',

na.action = na.omit,

lower = c(C0=0, f_k=0, k1=0, S0=0, k2=0),

upper = c(C0=60, f_k=0.999, k1=10,S0=60,k2=10))))

# get summary

info <- fits %>%

mutate(summary = map(fit, glance)) %>%

unnest(summary)

# get params

params <- fits %>%

mutate(., p = map(fit, tidy)) %>%

unnest(p)

# get confidence intervals

CI <- fits %>%

mutate(., cis = map(fit, confint2),

cis = map(cis, data.frame)) %>%

unnest(cis) %>%

rename(., conf.low = X2.5.., conf.high = X97.5..) %>%

group_by(., mcpa,rep) %>%

mutate(., term = c('C0', 'f_k','k1','S0','k2')) %>%

ungroup() %>%

select(., -data, -fit)

# merge parameters and CI estimates

params <- merge(params, CI, by = intersect(names(params), names(CI)))

# plot parameter estimates and confidence intervalls

params

# new data frame of predictions

new_preds <- dat %>%

do(., data.frame(time_d = seq(0, max(.$time_d), length.out = 300), stringsAsFactors = FALSE))

# create new predictions

preds2 <- fits %>%

mutate(., p = map(fit, augment)) %>%

unnest(p)

rename(., percent = .fitted)

# plot

p<-ggplot() +

geom_point(aes(time_d, percent, col = rep), size = 2, dat) +

geom_line(aes(time_d, .fitted, col = rep, group = rep), alpha = 0.5, preds2) +

facet_wrap(~ mcpa, labeller = labeller(.multi_line = FALSE)) +

scale_color_brewer(type='qual') +

theme_bw(base_size = 12) +

ylab('Cumulative 14C mineralization (%)') +

xlab('time (days)') +

theme(legend.position = c(0.9, 0.15))

p

# merge parameter values

C0s<-subset(params,term=="C0",select=c(mcpa,rep,estimate))

colnames(C0s)<-c('mcpa','rep','C0')

S0s<-subset(params,term=="S0",select=c(mcpa,rep,estimate))

colnames(S0s)<-c('mcpa','rep','S0')

f_ks<-subset(params,term=="f_k",select=c(mcpa,rep,estimate))

colnames(f_ks)<-c('mcpa','rep','f_k')

k1s<-subset(params,term=="k1",select=c(mcpa,rep,estimate))

colnames(k1s)<-c('mcpa','rep','k1')

k2s<-subset(params,term=="k2",select=c(mcpa,rep,estimate))

colnames(k2s)<-c('mcpa','rep','k2')

pars<-merge(C0s,S0s,by=c('mcpa','rep'))

pars<-merge(pars,k1s,by=c('mcpa','rep'))

pars<-merge(pars,k2s,by=c('mcpa','rep'))

pars<-merge(pars,f_ks,by=c('mcpa','rep'))

# define function to calculate half-live

f_hl <-function(t_hl,C0,S0,k1,k2,f_k){

y=-exp(k2*t_hl)+(exp(k1*t_hl)*(2*f_k*S0-2*S0)-2*f_k*S0)/(exp(k1*t_hl)*(f_k*(S0+C0)-S0-C0)+f_k*(-S0-C0)+2*C0)

}

f_hl <-function(t_hl,C0,S0,k1,k2,f_k){

y=exp(k2*t_hl)-(exp(k1*t_hl)*(2*f_k*S0-2*S0)-2*f_k*S0)/(exp(k1*t_hl)*(f_k*(S0+C0)-S0-C0)-f_k*(S0+C0)+2*C0)

}

# set start values for numerical estimation of half-lifes

pars<-arrange(pars,mcpa,rep)

pars$start=c(2,2,2,2,2,2,2,2,2,2,2,2,2,2,2,2,2,2,2,2,2)

# calculate half-lives

hl <- pars %>%

group_by(., mcpa, rep) %>%

nest() %>%

mutate(t_hl = purrr::map(data,

~nleqslv(.x$start, f_hl,control=list(btol=1e-2,maxit=1000,allowSingular=T),jacobian=FALSE,

method="Newton",xscalm="auto",C0=.x$C0,S0=.x$S0,k1=.x$k1,k2=.x$k2,f_k=.x$f_k)))

hl %>%

unnest(cols = "t_hl")

pars_final <- hl %>%

unnest(t_hl %>% map(~.x%>%data.frame()))

pars_final

pars_final <- hl %>%

mutate(., p = map(~.x, data.frame()))# %>%

data.frame(p)

unnest(p)

pars_final<-arrange(pars_final,mcpa,rep)

params <- fits %>%

mutate(., p = map(fit, tidy)) %>%

unnest(p)

# calculate mean half-lifes and plot half-lifes vs. initial conc.

t_hl_mean <- pars_final %>%

group_by(., mcpa) %>%

summarise(HL=mean(t_hl),HL_sd=sd(t_hl))

return(list("p"=p,"t_hl_mean"=t_hl_mean,"pars"=params))

}

f_1st_order<- function(dat){

# model fit

# fit over each set of groupings

fits <- dat %>%

group_by(., mcpa, rep) %>%

nest() %>%

mutate(fit = purrr::map(data, ~ nls_multstart(percent ~ C0*(1-exp(-k1*time_d)),

data = .x,

iter = 1000,

start_lower = c(C0=0, k1=0),

start_upper = c(C0=60, k1=10),

supp_errors = 'Y',

na.action = na.omit,

lower = c(C0=0, k1=0),

upper = c(C0=60,k1=10))))

info <- fits %>%

mutate(summary = map(fit, glance)) %>%

unnest(summary)

# get params

params <- fits %>%

mutate(., p = map(fit, tidy)) %>%

unnest(p)

# get confidence intervals

CI <- fits %>%

mutate(., cis = map(fit, confint2),

cis = map(cis, data.frame)) %>%

unnest(cis) %>%

rename(., conf.low = X2.5.., conf.high = X97.5..) %>%

group_by(., mcpa,rep) %>%

mutate(., term = c('C0', 'k1')) %>%

ungroup() %>%

select(., -data, -fit)

# merge parameters and CI estimates

params <- merge(params, CI, by = intersect(names(params), names(CI)))

# plot parameter estimates and confidence intervalls

params

# new data frame of predictions

new_preds <- dat %>%

do(., data.frame(time_d = seq(0, max(.$time_d), length.out = 300), stringsAsFactors = FALSE))

# create new predictions

preds2 <- fits %>%

mutate(., p = map(fit, augment)) %>%

unnest(p)

rename(., percent = .fitted)

# plot

p<-ggplot() +

geom_point(aes(time_d, percent, col = rep), size = 2, dat) +

geom_line(aes(time_d, percent, col = rep, group = rep), alpha = 0.5, preds2) +

facet_wrap(~ mcpa, labeller = labeller(.multi_line = FALSE)) +

scale_color_brewer(type='qual') +

theme_bw(base_size = 12) +

ylab('Cumulative 14C mineralization (%)') +

xlab('time (days)') +

theme(legend.position = c(0.9, 0.15))

p

# merge parameter values

C0s<-subset(params,term=="C0",select=c(mcpa,rep,estimate))

colnames(C0s)<-c('mcpa','rep','C0')

k1s<-subset(params,term=="k1",select=c(mcpa,rep,estimate))

colnames(k1s)<-c('mcpa','rep','k1')

pars<-merge(C0s,k1s,by=c('mcpa','rep'))

# calculate half-lifes

pars_final <- pars %>%

mutate(t_hl = log(2)/k1)

pars_final<-arrange(pars_final,mcpa,rep)

# calculate mean half-lifes and plot half-lifes vs. initial conc.

t_hl_mean <- pars_final %>%

group_by(., mcpa) %>%

summarise(HL=mean(t_hl),HL_sd=sd(t_hl))

ggplot(t_hl_mean,aes(x=mcpa,y=HL))+

geom_point(size=3)+

geom_errorbar(aes(ymin=HL-HL_sd,ymax=HL+HL_sd),width=0.2)+

xlab('Initial MCPA concentration (?g kg-1)')+

ylab('Half-life (d)')

return(list("p"=p,"t_hl_mean"=t_hl_mean,"pars"=params))

}

f_without_2nd_pool<-function(dat){

# model fit

# fit over each set of groupings

fits <- dat %>%

group_by(., mcpa, rep) %>%

nest() %>%

mutate(fit = purrr::map(data, ~ nls_multstart(percent ~ C0-C0/((1-f_k)*exp(k1*time_d)+f_k),

data = .x,

iter = 1000,

start_lower = c(C0=0, f_k=0, k1=0),

start_upper = c(C0=60, f_k=0.999, k1=10),

supp_errors = 'Y',

na.action = na.omit,

lower = c(C0=0, f_k=0, k1=0),

upper = c(C0=60, f_k=0.999, k1=10))))

# get summary

info <- fits %>%

mutate(summary = map(fit, glance)) %>%

unnest(summary)

# get params

params <- fits %>%

mutate(., p = map(fit, tidy)) %>%

unnest(p)

# get confidence intervals

CI <- fits %>%

mutate(., cis = map(fit, confint2),

cis = map(cis, data.frame)) %>%

unnest(cis) %>%

rename(., conf.low = X2.5.., conf.high = X97.5..) %>%

group_by(., mcpa,rep) %>%

mutate(., term = c('C0', 'k1','f_k')) %>%

ungroup() %>%

select(., -data, -fit)

# merge parameters and CI estimates

params <- merge(params, CI, by = intersect(names(params), names(CI)))

params

# new data frame of predictions

new_preds <- dat %>%

do(., data.frame(time_d = seq(0, max(.$time_d), length.out = 300), stringsAsFactors = FALSE))

# create new predictions

preds2 <- fits %>%

mutate(., p = map(fit, augment)) %>%

unnest(p)

# plot

p<-ggplot() +

geom_point(aes(time_d, percent, col = rep), size = 2, dat) +

geom_line(aes(time_d, percent, col = rep, group = rep), alpha = 0.5, preds2) +

facet_wrap(~ mcpa, labeller = labeller(.multi_line = FALSE)) +

scale_color_brewer(type='qual') +

theme_bw(base_size = 12) +

ylab('Cumulative 14C mineralization (%)') +

xlab('time (days)') +

theme(legend.position = c(0.9, 0.15))

p

# merge parameter values

C0s<-subset(params,term=="C0",select=c(mcpa,rep,estimate))

colnames(C0s)<-c('mcpa','rep','C0')

f_ks<-subset(params,term=="f_k",select=c(mcpa,rep,estimate))

colnames(f_ks)<-c('mcpa','rep','f_k')

k1s<-subset(params,term=="k1",select=c(mcpa,rep,estimate))

colnames(k1s)<-c('mcpa','rep','k1')

pars<-merge(C0s,k1s,by=c('mcpa','rep'))

pars<-merge(pars,f_ks,by=c('mcpa','rep'))

# calculate half-lifes

pars_final <- pars %>%

mutate(t_hl = 1/k1*log(1/(1-f_k)+1))

pars_final<-arrange(pars_final,mcpa,rep)

# calculate mean half-lifes and plot half-lifes vs. initial conc.

t_hl_mean <- pars_final %>%

group_by(., mcpa) %>%

summarise(HL=mean(t_hl),HL_sd=sd(t_hl))

return(list("p"=p,"t_hl_mean"=t_hl_mean,"pars"=params))

}

# FULL model

# select only data of experiments with 30-500 ?g kg-1 MCPA

dat1 <-subset(dat,mcpa %in% c(30,50,100,500))

out_list<-f_full(dat1)

out_list$p

t_hl_mean<-out_list$t_hl_mean

pars<-out_list$pars

# SIMPLIFICATION to first-order kinetics

# select only data of experiments with 1000 and 5000 ?g kg-1 MCPA

dat2 <-subset(dat,mcpa %in% c(1000,5000))

out_list<-f_1st_order(dat2)

out_list$p

t_hl_mean<-rbind(t_hl_mean,out_list$t_hl_mean)

pars<-rbind(pars,out_list$pars)

# SIMPLIFICATION by neglecting second 14C pool (mineralizable 14C-SOM)

# select only data of experiments with 20000 ?g kg-1 MCPA

dat3 <-subset(dat,mcpa %in% c(20000))

out_list<-f_without_2nd_pool(dat3)

out_list$p

t_hl_mean<-rbind(t_hl_mean,out_list$t_hl_mean)

pars<-rbind(pars,out_list$pars)

View(pars)

ggplot(pars,aes(x=mcpa,y=estimate))+

#geom_jitter(aes(colour=rep),size=4)+

geom_pointrange(aes(ymin=estimate-std.error,ymax=estimate+std.error,colour=rep),

position=position_jitter(width=0.2),size=1)+

facet_wrap(~term)+

xlab('Initial MCPA concentration (?g kg-1)')+

ylab('Parameter value')+

theme_bw()+

theme(axis.text=element_text(size=16),axis.title=element_text(size=18,face="bold"))

THEME <- theme(panel.border = element_rect(colour = "black", fill=NA),

axis.text = element_text(colour = 1, size = 12),

legend.background = element_rect(linetype = 2, size = 0.5, colour = 1),

axis.title.x = element_text(size = 14),

axis.title.y = element_text(size = 14),

panel.grid = element_blank(),

strip.text.x = element_text(size = 14),

strip.text.y = element_text(size = 14))

Halbwertszeit_sd<-left_join(Halbwertzeit,t_hl_mean, by = c('mcpa'='mcpa'))

?stat_compare_means

hlf<-ggplot(Halbwertszeit_sd,aes(x=mcpa,y=emmean))+

geom_point(size=2, alpha =.6)+

geom_errorbar(aes(ymin=emmean-HL_sd,ymax=emmean+HL_sd),width=0.1)+

labs(x = expression(Initial~MCPA~concentration~(ug~kg^-1)),

y = expression(paste("Half-life (d)"))) +

theme_bw()+

THEME

hlf

ggsave("half-life time.tiff",hlf, dpi = 300, width = 6, height = 2.5)
